# Simulated microgravity triggers epithelial mesenchymal transition in human keratinocytes

**DOI:** 10.1038/s41598-017-00602-0

**Published:** 2017-04-03

**Authors:** Danilo Ranieri, Sara Proietti, Simona Dinicola, Maria Grazia Masiello, Benedetta Rosato, Giulia Ricci, Alessandra Cucina, Angela Catizone, Mariano Bizzarri, Maria Rosaria Torrisi

**Affiliations:** 1 Dipartimento di Medicina Clinica e Molecolare, Sapienza Università di Roma, Rome, Italy; 2 Dipartimento di Medicina Sperimentale, Seconda Università di Napoli, Napoli, Italy; 3 Dipartimento di Chirurgia “P. Valdoni”, Sapienza Università di Roma, Rome, Italy; 4 Dipartimento di Scienze Anatomiche, Istologiche, Medico Legali e dell’Apparato Locomotore, Sapienza Università di Roma, Rome, Italy; 5 Dipartimento di Medicina Sperimentale, Sapienza Università di Roma, Rome, Italy

## Abstract

The microgravitational environment is known to affect the cellular behaviour inducing modulation of gene expression and enzymatic activities, epigenetic modifications and alterations of the structural organization. Simulated microgravity, obtained in the laboratory setting through the use of a Random Positioning Machine (RPM), represents a well recognized and useful tool for the experimental studies of the cellular adaptations and molecular changes in response to weightlessness. Short exposure of cultured human keratinocytes to the RPM microgravity influences the cellular circadian clock oscillation. Therefore, here we searched for changes on the regenerative ability and response to tissue damage of human epidermal cells through the analysis of the effects of the simulated microgravity on the re-epithelialization phase of the repair and wound healing process. Combining morphological, biochemical and molecular approaches, we found that the simulated microgravity exposure of human keratinocytes promotes a migratory behavior and triggers the epithelial-mesenchymal transition (EMT) through expression of the typical EMT transcription factors and markers, such as Snail1, Snail2 and *ZEB2*, metalloproteases, mesenchymal adhesion molecules and cytoskeletal components.

## Introduction

Microgravity is known to affect many physiological functions on the exposed organisms, which react to the altered environment adapting the tissue and cellular behavior. When compared to the on-ground force of gravity (1*g*), the microgravity conditions are able to induce a variety of alterations of the structural organization of the cells consequent to epigenetic modifications as well as modulation of gene expression and enzymatic activities^1–8^. Simulated microgravity, obtained in the laboratory setting through the use of a Random Positioning Machine (RPM), represents a well recognized and useful tool for the experimental studies of the cellular adaptations and molecular alterations induced by the weightlessness stimulus^9^.

The human skin is a peripheral tissue highly exposed to environmental changes and its epidermal keratinocyte components are known to be characterized by the presence of a functional molecular clock^10, 11^. We have very recently shown that short exposure to the simulated microgravitational environment influences the circadian clock oscillation of human keratinocytes, without interfering with the actin cytoskeleton organization and the proliferation or apoptotic rate^12^. These results have been interpreted in light of the existence of an integrated signaling network coupling mechano-sensitive pathways to gene regulation and transcription^13^. Consistent with this hypothesis, the expression profiles of many different genes of human keratinocytes are affected under exposure to simulated microgravity in a time-dependent manner and during recovery^14^. In addition, it has been recently reported that long term exposure to microgravity conditions is able to affect mouse skin homeostasis^15, 16^, mostly inducing expression modulations of those genes responsible for remodeling of the extracellular matrix^15^ and leading to dermal atrophy^16^: however, the physiological behaviour of the epidermal keratinocytes and their molecular pattern did not appear significantly altered in such environment^16^. Therefore, in the present paper, we searched for changes on the regenerative ability and response to tissue damage of human epidermal cells through the analysis of the effects of the simulated microgravity on the repair process. Combining different morphological and molecular approaches, we found that the simulated microgravity exposure of human keratinocytes may affect tissue repair promoting the re-epithelialization phase of the wound healing process, triggering the complex set of events defined epithelial-mesenchymal transition (EMT), in which the epithelial cells acquire a typical migratory phenotype of mesenchymal cells due to reprogramming of gene expression and appearance of specific markers^17–19^.

## Results

### Simulated microgravity changes the migratory behavior of human keratinocytes

**Figure 1:**
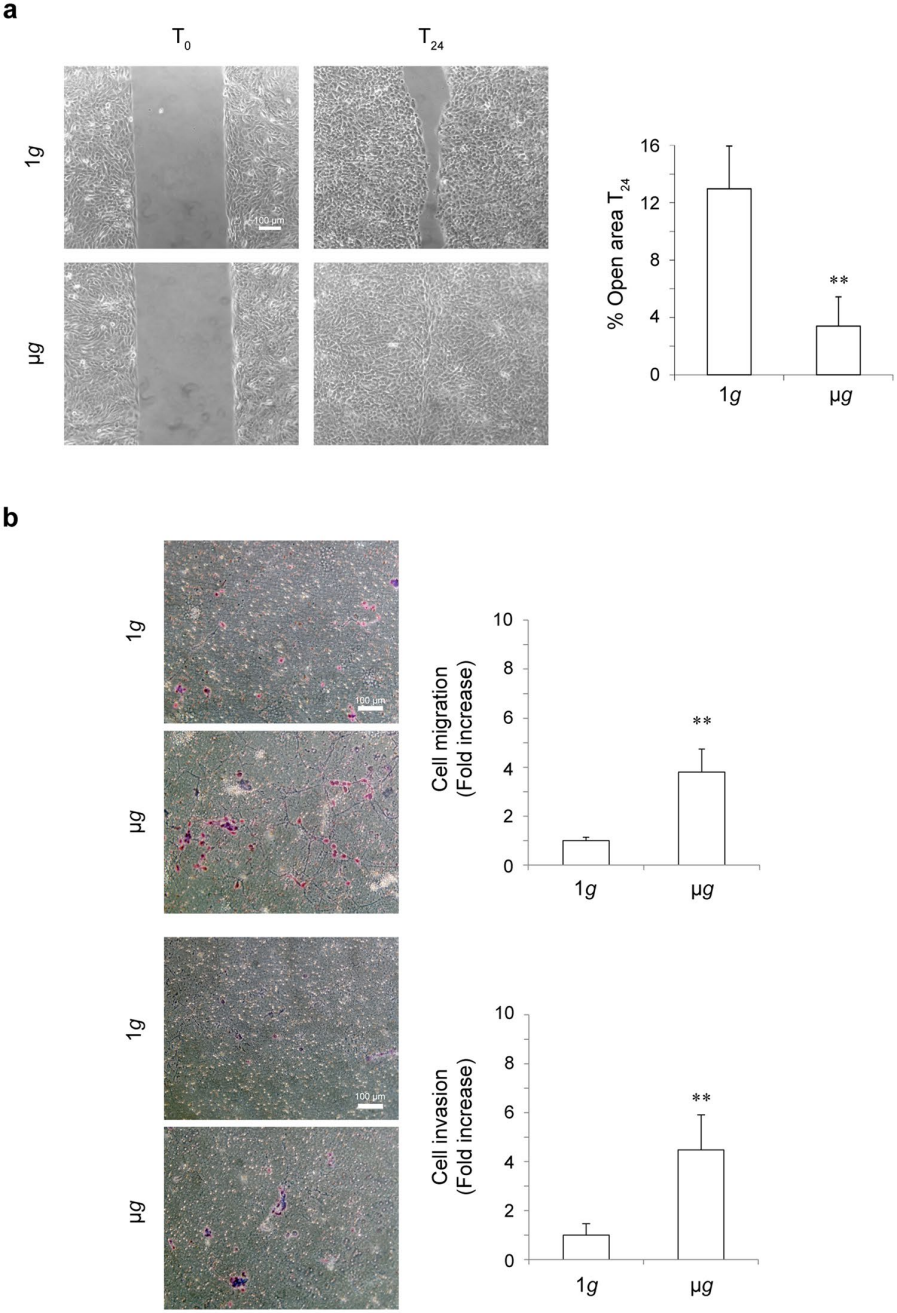
Simulated microgravity exposure enhances cell migration and induces invasiveness in human keratinocytes. (**a**) HaCaT cells were grown until confluence and allowed to migrate for 24 hours (T_24_) under simulated microgravity (μ*g*) or normal gravity (1*g*) in a cell-free scratch area. The closure of the scratch area is higher in cells exposed to μ*g* respect to cells kept at 1*g*. Cell migration was calculated as reported in methods (n = 3). (**b**) HaCaT cells were kept at 1*g* or exposed to μ*g* for 24 hours. Cells were then seeded on uncoated (upper panels) or matrigel pre-coated (lower panels) transwell Boyden chambers and complete medium was added in the bottom chamber for chemotactic stimulation for 24 hours. Cell migration (upper panels) and invasion (lower panels) are significantly increased in μ*g* cultures compared to control cells. Quantitative analysis was assessed as reported in methods and results expressed as mean ± standard deviation (SD) (n-4). Student’s *t* test was performed and significance level has been defined as follows: **p < 0.001 vs the control cells (1*g*).

Because the wound healing process requires the proliferative and migratory activity of the keratinocytes and it has been proposed that the expression of several growth factors and cytokines is modulated by microgravity upon wound repair^20^, here we analyzed in detail the possible effects of simulated microgravity on the motility and repairing properties of human keratinocytes. To this aim, we used human immortalized HaCaT keratinocytes, a widely utilized cell model to study epidermal physiology^21, 22^, used also in a very recent study of our group focused on the effects of microgravity on the circadian clock^12^. Here we evaluated first the impact of simulated microgravity exposure on the spontaneous keratinocyte motility using the “scratch assay”. The scratch was performed on confluent HaCaT cultures and cells were exposed to simulated microgravity on the RPM or kept at 1*g* as control (Fig. 1a). After 24 hours from the scratch we observed an enhancement of cell migration during simulated microgravity treatment respect to the cells kept at 1*g* (Fig. 1a). Automated analysis using TScratch software clearly showed differences in migration properties calculated as percentage of the open surface residual area respect to 100% (300 μm) at the initial time points T_0_ of each condition (1*g* or μ*g*): in fact, in simulated microgravity exposed cells, the percentage of the open surface area was much lower compared to control cells (3% Vs 12%; ** *p* < 0.001).

Then we investigated the migration induced by chemotactic stimulation under simulated microgravity. To this aim we analyzed the capacity of HaCaT cells, kept at 1*g* or exposed to simulated microgravity for 24 hours, to migrate through transwell Boyden chamber filters when complete medium was added in the bottom chamber for chemotactic stimulation. The results showed an increase of cell migration, evaluated by counting the cell number/field, in the chambers exposed to simulated microgravity compared to control (Fig. 1b, top panels). Thus, simulated microgravity exposure induced keratinocyte cell migration. Based on this finding, we investigated the possible effect of simulated microgravity in triggering not only migration but invasive ability of the HaCaT cells which are known to be non invasive^21^. To this aim we analyzed if simulated microgravity exposed cells as above could migrate through the transwell Boyden chambers pre-coated with a thin layer of matrigel, a gel composed of reconstituted basement membrane elements resembling the basement membrane *in vivo*. The results, evaluated as above, showed detection of cell invasion only in simulated microgravity exposed keratinocytes (Fig. 1b, bottom panels).

Taken together our results revealed that simulated microgravity exposure was able to enhance the migration and to trigger the invasion of human keratinocytes.

### The proliferation or apoptosis of the keratinocytes are not affected by the exposure to the simulated microgravity

In order to better clarify our results on the migratory and invasive effects of the simulated microgravity, we wanted to rule out the possibility that cell proliferation or apoptosis could contribute to the repopulation of the wounded area. The growth ability of the simulated microgravity treated cells respect to those kept at 1*g* was assessed at the same time point used for the motility experiments. The proliferation assay showed no significant differences in the increase of cell number at 24 hours upon simulated microgravity exposure respect to control cells (Supplementary Fig. S1a). We evaluated also the cell cycle in simulated microgravity treated cellsand controls: cytofluorimetric analysis showed that the cell cycle was not significantly influenced by the exposure as shown by the percentage of cells in G0/G1, S and G2/M phases (Supplementary Fig. S1a). To exclude also the possible contribution of changes in cell apoptosis in our experiments, the Annexin V assay has been done. As shown in Supplementary Fig. S1a, bottom panels, the apoptotic rates induced by camptothecin treatment, used as positive control, were similar in cells exposed to simulated microgravity or kept at 1*g*. In addition, mitotic spindles and midbodies were counted along the entire z axis of equivalent areas occupied by the cells in control and simulated microgravity conditions, either in areas where cells were confluent or in regions of the scratch gap (Supplementary Fig. S1b). Again, we did not detect significant differences in the number of mitotic cells/fields in all cultural conditions (Supplementary Fig. S1b). These findings clearly indicate that the more rapid wound closure on simulated microgravity environment was not due to a major proliferative activity or decreased apoptotic rate, but to an enhanced migratory capacity.

### Simulated microgravity induces the cytoskeletal reorganization required for cell migration

**Figure 2:**
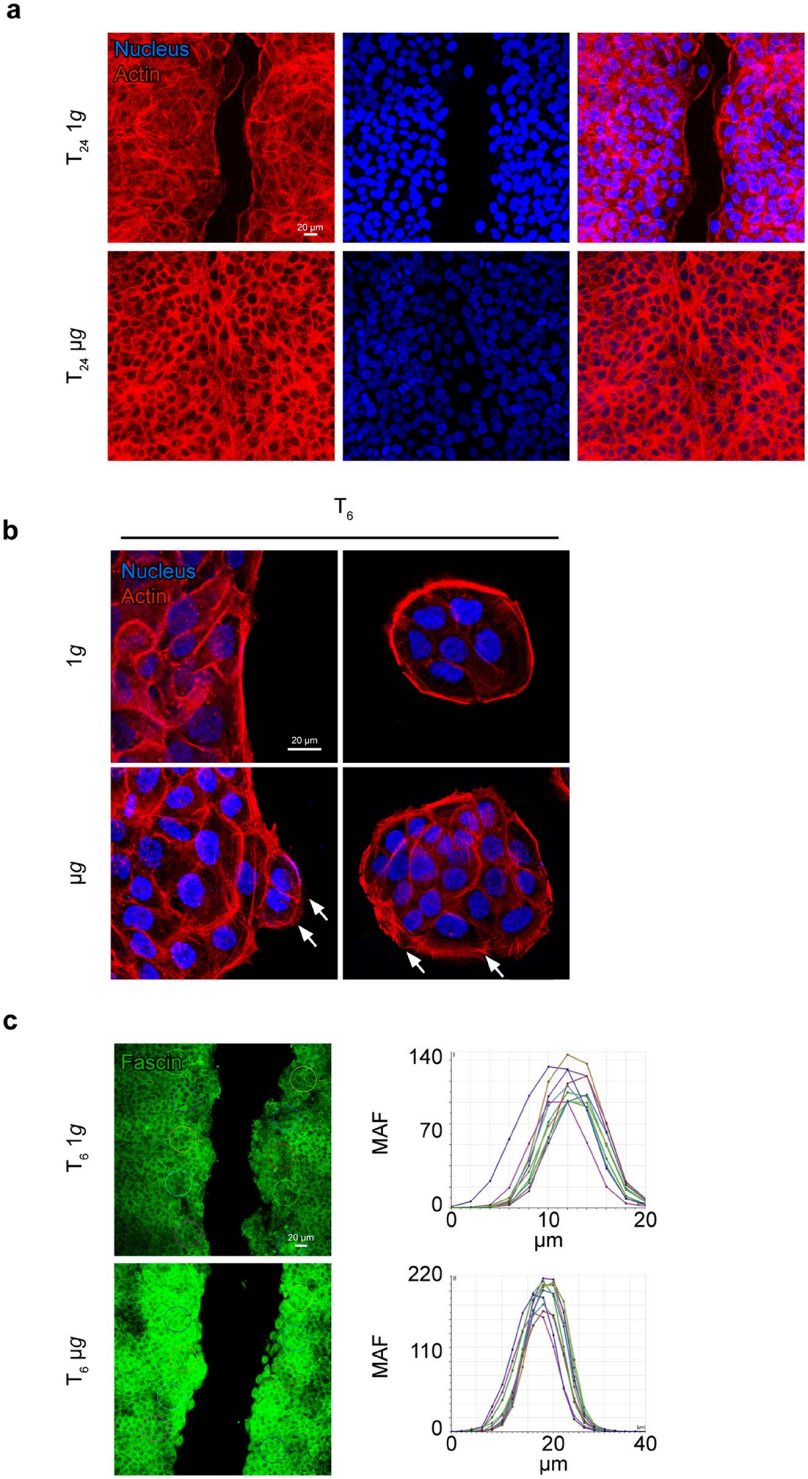
Simulated microgravity exposure affects keratinocyte growth mode and their morphology during migration. (**a**) Fluorescence analysis using TRITC-phalloidin, to visualize the F-actin cytoskeleton, on the scratch assay performed as in Fig. 1, confirms that in μ*g* cultures the scratch area is sealed, while only few migrating cells are visible in the corresponding control cultures. (**b**) Fluorescence performed as above in cells grown until semi-confluence and exposed to μ*g* for 6 hours shows that, in control cultures, cells maintain an actin cytoskeleton organization in peripheral cortical bundles (top panels); in μ*g*-treated cultures, lamellipodia and ruffles are frequently observed in peripheral cells (bottom panels, arrows). (**c**) Scratch assay was performed as reported above and cells were left to migrate for 6 hours. Quantitative immunofluorescence analysis, expressed as Max Amplitude Fluorescence (MAF), shows that fascin is increased in μ*g* exposed cells compared to controls.

To investigate the cell morphology and actin cytoskeleton during the keratinocyte migration when occurring under simulated microgravity, fluorescence analysis was performed in cells exposed to simulated microgravity or kept at 1*g* as a control using TRITC-conjugated phalloidin, to visualize the F-actin cytoskeleton organization on the scratch test as above. The results confirmed that, in simulated microgravity treated cells at 24 hours from the scratch, the wound area was completely sealed, while only sparse migrating cells were evident in the wound area of 1*g* cells (Fig. 2a). However, to appreciate in detail the actin cytoskeletal organization, the phalloidin fluorescence was performed also on cells exposed to simulated microgravity for 6 hours and grown in conditions of semi-confluence. Fluorescence analysis showed that the organization of the cells in untreated conditions remained, as expected, cobblestone-shaped, maintaining the actin cytoskeleton distribution in peripheral cortical bundles characteristic of the cultured epidermal keratinocytes (Fig. 2b, top panels). In simulated microgravity exposed cells, in addition to the cortical bundles, lamellipodia and ruffles were frequently observed, particularly in the cells located at the periphery of the colonies (Fig. 2b lower panels, arrows).

The observed protrusions are known to be formed by an actin assembling process involving many proteins such as the fascin, a protein essential for the generation and maintenance of tight F-actin bundles of filopodia and to cell migration^23^. Therefore, immunofluorescence analysis of fascin staining was performed and quantitative evaluation of the signal intensity was assessed: the results clearly indicated an increase of fascin expression in cells exposed to simulated microgravity in comparison to control cells (Fig. 2c). Taken together, our findings showed that simulated microgravity was able to cause, in human keratinocytes under the scratch assay, the appearance of morphological features and cytoskeletal reorganization consistent with the migratory and invasive behavior.

### Modulation of EMT markers is induced by simulated microgravity

Since the triggering of migratory and invasive properties involves a process named epithelial mesenchymal transition (EMT), we wondered whether the phenotypic changes observed under simulated microgravity exposure would be associated to the expression of well recognized epithelial and mesenchymal biomarkers of EMT.

**Figure 3:**
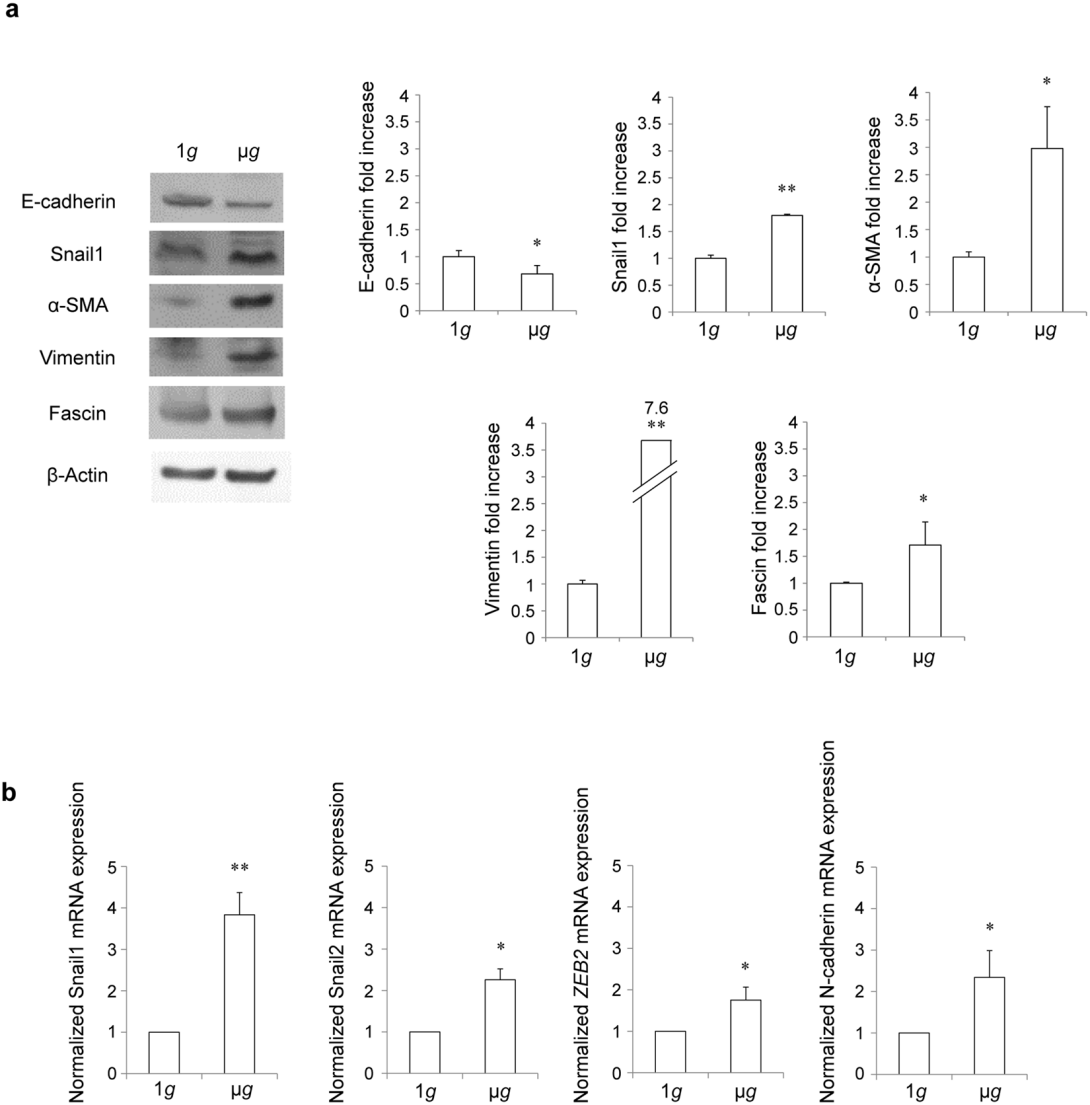
Simulated microgravity induces EMT in keratinocytes. HaCaT cells were exposed to μ*g* or kept at 1*g* for 24 hours. (**a**) Western blot analysis shows, in μ*g* cells, a reduction of the epithelial marker E-cadherin and an increase of fascin and of the mesenchymal markers α-SMA and vimentin as well as of the EMT-related transcription factor Snail1. The equal loading was assessed using anti-β actin antibody. For the densitometric analysis, the values were normalized, expressed as fold increase and reported in graph as mean value ± SD (n = 3). (**b**) RT-PCR in cells treated as above shows that the mRNA transcript levels of Snail1, Snail2 and *ZEB2* and N-cadherin are increased in simulated microgravity-treated cells compared to controls. Results are expressed as mean value ± SD (n = 3). Student’s *t* test was performed and significance levels have been defined as follows: *p < 0.05 vs 1 *g* cells; **p < 0.001 vs 1*g* cells.

First, we investigated by western blot analysis the modulation of the expression of the cell-cell adhesion molecule E-cadherin and of the cytoskeletal components α-SMA, vimentin and fascin. The results clearly showed a reduction of the epithelial marker E-cadherin and the appearance of typical mesenchymal markers like vimentin and α-SMA in simulated microgravity treated cells respect to cells kept at 1g (Fig. 3a). The evaluation of E-cadherin was further supported by the observed decrease of E-cadherin fragment, resulting from the cleavage of E-cadherin (Supplementary Fig. S2a). In agreement with the immunofluorescence results reported above, the protein expression of fascin was increased in cells exposed to simulated microgravity compared to control cells (Fig. 3a).

During the EMT process, the gene reprogramming required to obtain the mesenchymal-like phenotype involves several transcription factors^19, 24^. One of the most important transcription factor involved in EMT induction is Snail1, associated with the initiation of the process^25^. In simulated microgravity exposed cells, Snail1 appeared to be expressed at both protein and mRNA levels more than in control cells (Fig. 3a,b). In addition, we evaluated by RT-PCR other biomarkers involved in EMT: the transcription factors positively regulated during the EMT process, Snail2 and *ZEB2*, were more expressed in simulated microgravity treated cells respect to the control cells. The transcript levels of the mesenchymal protein N-cadherin, which is generally induced in its expression during EMT, were higher in the simulated microgravity treated cells compared with cells kept at 1*g* (Fig. 3b). Taken all together these results strongly suggest that simulated microgravity was capable to trigger EMT in keratinocytes.

**Figure 4:**
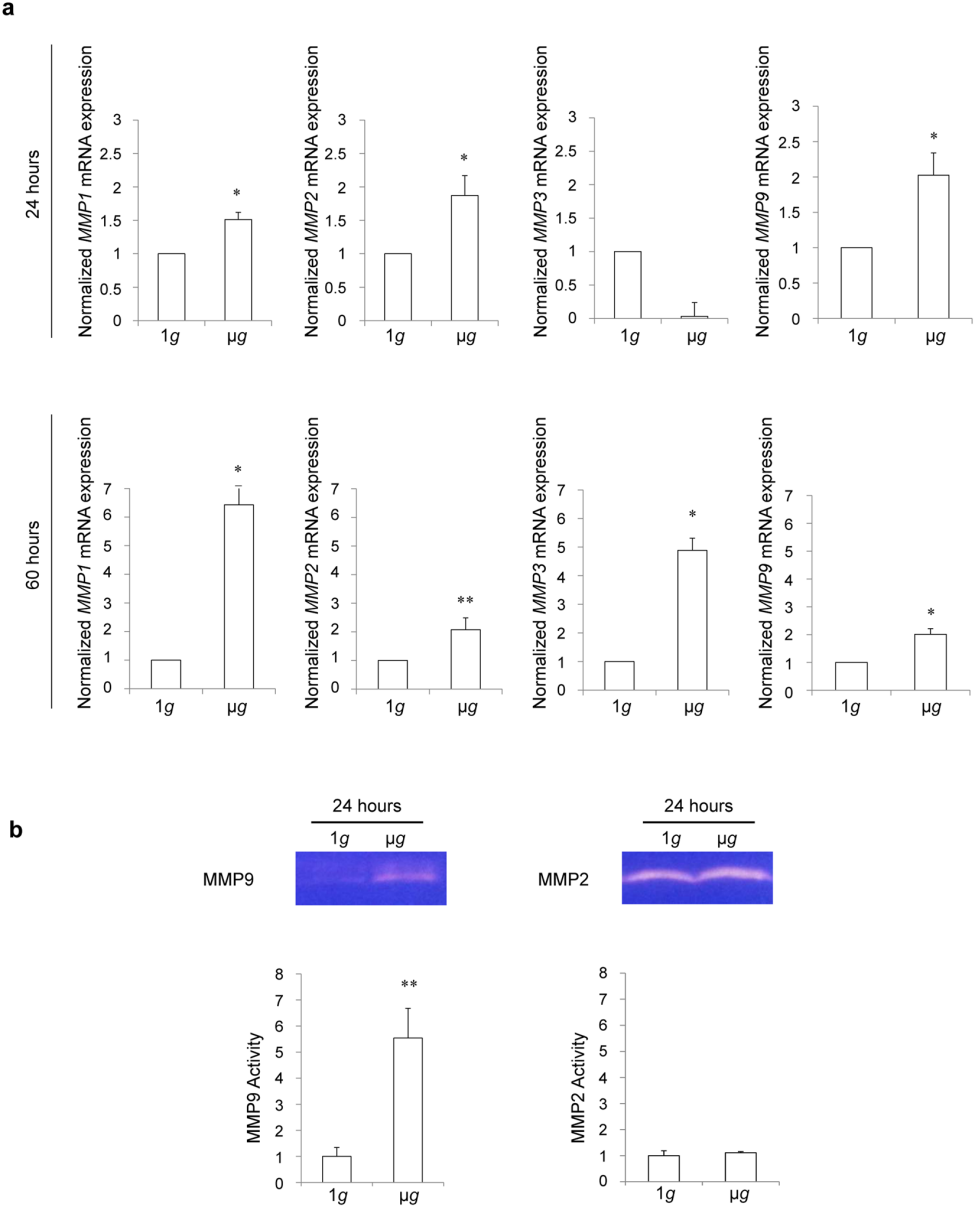
Simulated microgravity exposure modulates the expression and the enzymatic activity of metalloproteases. HaCaT cells were kept at 1 *g* or exposed to simulated microgravity for 24 hours (a, b) or 60 hours (a). (**a**) In cells exposed to μ*g* for 24 hours, RT-PCR shows an increase for *MMP1*, *MMP2* and *MMP9*, but a decrease for *MMP3* mRNA transcript levels; after 60 hours of μ*g* exposure, all the MMPs are increased if compared to 1*g*. Results are expressed as mean ± SD (n = 3). (**b**) The images of the Gelatin zymography analysis are cropped and show that the enzymatic activity of *MMP9*, but no that of *MMP2*, is increased in μ*g* cells compared to controls. Results are expressed as mean ± SD (n = 3). Student’s *t* test was performed and significance levels have been defined as follows: *p < 0.05 vs 1*g* cells; **p < 0.001 vs 1*g* cells.

The metalloproteases (MMPs), which play a key role in wound healing, are known to be expressed by migrating and proliferating keratinocytes during the re-epithelization process. In particular, *MMP1*, *MMP2* and *MMP9* expression and release enhance the invasive properties of the cells^26, 27^. In order to evaluate the mRNA expression, RT-PCR was performed on cells treated for 24 and 60 hours in simulated microgravity and at 1*g*: at 24 hours, our results showed an increase of mRNA expression for *MMP1*, *MMP2* and *MMP9* but a decrease for *MMP3*. However, after 60 hours of exposure, the transcript levels of all the metalloproteases measured (*MMP1*, *MMP2*, *MMP3*, *MMP9*) were increased compared with those of cells kept at 1*g* (Fig. 4a). A further confirmation of what has been observed in RT-PCR experiments was obtained by western blot analysis of the protein expression of *MMP1* and MMP3, showing an increase in cells exposed to simulated microgravity respect to control cells (Supplementary Fig. S2b). Since metalloproteases are highly regulated not only in their expression, but mostly in their enzymatic activity, which can also be controlled by proteinase inhibitors, we evaluated by zymography the activities of *MMP2* and *MMP9* in cells treated as above. Although we found a significant increase of enzyme activity only for *MMP9* in cells exposed to simulated microgravity compared to controls (Fig. 4b), our findings may suggest that microgravity was able to induce enhanced degradation of the extracellular matrix required for the cell motility.

## Discussion

Wounds of the skin are known to generate a mechanical stretch on the marginal epidermal keratinocytes and dermal fibroblasts are able to trigger the healing response through activation of mechano-signal transduction^28^. Microgravity exposure affects the cell behavior changing the mechanical environment and, therefore, it allows to study the response to mechanical forces and their signaling^29^. Thus, we wondered if the mechanical environment induced by simulated microgravity would affect the repair and wound healing process through the acquisition by the keratinocytes of a motile and invasive mesenchymal phenotypes. We showed that simulated microgravity exposure of human keratinocytes is able to enhance cell migration either spontaneous, as assessed by the migration assay, or induced by chemotactic stimulation through the filter pores of transwell Boyden chambers. These results not only are consistent with the published observations that keratinocyte migration is improved under mechanical stretch^30^, but also reveal that this migratory response is the consequence of the expression of some genes, such as Snail1, Snail2 and *ZEB2*, and transcriptional regulation of others, such as those of *MMP1*, *MMP2*, *MMP9* and N-cadherin, which are characteristics of the EMT process. Complete gene expression profiling in human keratinocytes has been previously reported and showed extensive transcriptional up- or down-regulation induced by simulated microgravity^14^: however, comparing to our present results, that analysis pointed only to the clusters of genes more differentially modulated and the length of exposure, starting from 3 days, was longer than in our experimental conditions^14^.

The reorganization of the cell cytoskeleton is known to be altered by both real and simulated microgravity in many human cell types, including chondrocytes^31, 32^ and endothelial cells^4^, and different cytoskeletal proteins, components of the intermediate filaments, actin bundles and microtubules, are changed in their expression under microgravity^4, 31, 32^. Consistent with those studies, our results showed that simulated microgravity was able to cause, in human keratinocytes under the scratch assay, the acquisition of morphological features and cytoskeletal reorganization consistent with the migratory and invasive behavior, such as the appearance of α-SMA and vimentin, usually absent in epithelial cells, as well as the enhanced expression of fascin, a protein essential for the filopodia structure and for cell migration^23^.

The exposure of cells to decreased gravity is known to induce alterations of the biological behavior affecting proliferation and apoptosis^33–35^, but we have shown in a previous study of our group that, at least in the cell model used and in the experimental condition tested, the proliferative and apoptotic rates were similar in cells exposed to simulated microgravity compared to 1*g* ^12^. Here we showed again that the proliferation or apoptosis of the keratinocytes did not appear to be affected by the simulated microgravity environment in our experimental conditions, further indicating that the more rapid scratch closure could not be ascribed to a major proliferative activity or decreased apoptotic rate, but to a real enhanced migratory capacity.

The EMT process is a starting event in wound healing and involves the cellular acquisition of a motile mesenchymal phenotype^17, 18^. We have recently reported that, in both HaCaT cells and primary cultured human keratinocytes, EMT can be triggered by the isoform switching of the fibroblast growth factor receptor 2 (*FGFR2*)^36, 37^, a key regulator of the re-epithelialization during wound healing^38^. Therefore, we might propose that the migratory ability and the cytoskeletal reorganization required for cell migration, as observed in our present *in vitro* study, would presumably correspond to a stimulatory effect of the microgravity on the *in vivo* wound healing.

Finally, prolonged exposure to altered gravity of mice kept aboard the International Space Station (ISS) and extensive transcriptomic analysis of their skin samples have shown that the major cutaneous alteration is the dermal atrophy, possibly due to an imbalance between synthesis and degradation of extracellular matrix components^16^. Since the dermal fibroblasts, which are responsible for matrix deposition and degradation, are finely regulated in their function by several paracrine mediators released by the epidermal cells, we may suggest that the microgravity-triggered EMT of the keratinocytes, occurring shortly after exposure, would affect at longer times the dermal homeostasis and its adaptive response.

## Methods

### Cells and treatments

The human keratinocyte cell line HaCaT was cultured in Dulbecco’s Modified Eagles Medium (DMEM) supplemented with 10% Fetal Bovine Serum (FBS) plus antibiotics. Cells were seeded in Opticell units (Biocrystal Ltd, Westerville, OH, USA) and allowed to attach on both the two inner membrane surfaces of the chamber by repetitive turning upside down every 15 min during 4 hours. All the experiments were performed after 24 hours from cell plating and cells were not synchronized. Simulated microgravity conditions were obtained by positioning the Opticell chambers containing HaCaT cells in the Random Positioning Machine (RPM) device (Dutch Space, Leiden, Netherlands) inside a humidified incubator (5% CO_2_ at 37 °C), setting the angular velocity of rotation at 90°/s as maximum and 30°/s as minimum, in a random mode^1^. Under this experimental condition, the cells were exposed to simulated microgravity conditions ranging from 0 to 0.01*g*, and experiments performed for 6, 24 or 60 hours.

### Scratch assay

The scratch assay was performed using special double well culture inserts (ibidi GmbH, Martinsried, Germany). Each insert was placed in a well of a 8-well μ-slide (ibidi) and 70 μl of cell suspension (prepared at the concentration of 4.5 × 10^5^ cells/ml) were placed into both well of each insert. At confluence, the culture inserts were gently removed and cells were fed with complete medium. Slides were then exposed to simulated microgravity condition (μ*g*) or kept on ground as control (1*g*) for 24 hours. Cells were photographed at 10X magnification with a Nikon DS-Fi1 camera (Nikon Corporation, Tokyo, Japan), coupled with a Zeiss Axiovert optical microscope (Zeiss, Oberkochen, Germany) and the mean percentage of residual open area compared to the respective cell-free surface in T_0_ using TScratch software^39^. Four replicate inserts were used for each experimental conditions and each experiment was performed in triplicate.

### Cell migration assay

Cells were treated for 24 hours in μ*g* or kept at 1*g* then were placed (5 × 10^3^ cells) in the upper side of 8-μm filters (Falcon, BD Biosciences) (upper chamber) in 0.5 ml DMEM 0.1% FBS. These filters were placed in a 24-well plate (Falcon, BD Biosciences, San Jose, CA, USA) (lower chamber), containing 0.8 ml DMEM 10% FBS. Chambers were kept in an incubator for 24 hours. After incubation, cells from the upper surface of filters were removed and the migratory cells on the lower surface of membranes were fixed and stained with hematoxylin/eosin. The membranes were examined microscopically, and cell migration was determined by counting the number of cells on membranes in at least 4–5 randomly selected fields 10X magnification using a Zeiss Axiovert 10 optical microscope. Cellular migration value was reported as mean ± standard deviation (SD) of migrating cells/field count and normalized to control cells. For each experimental conditions, four independent experiments in duplicate were performed.

### Cell invasion assay

Cells were treated for 24 hours in μ*g* or kept at 1*g* and then placed in the BD Bio-Coat^TM^ growth factor reduced MATRIGEL^TM^ invasion chamber (Falcon, BD Biosciences) as reported^40^. Cellular invasion value was reported as mean ± SD of migrating cells/field count and normalized to control cells. Four independent experiments in duplicate were performed.

### Cell proliferation assay

Cells were treated for 24 hours in μ*g* or kept at 1*g* and then counted with a particle count and size analyzer (Beckman-Coulter, Inc., Fullerton, CA, USA) and by a Thoma hemocytometer. Three independent experiments in duplicate were performed.

### Flow cytometry

For apoptotic cell death assay with Annexin V and 7-aminoactinomycine-D (7-AAD) staining, cells were treated as described^40^. For apoptosis induction cells were treated for 24 hours with camptothecin 1 μM (Sigma-Aldrich, St Louis, MO, USA, C9911). For cell cycle analysis cells were treated and analyzed as reported^41^. Three independent experiments in duplicate were performed.

### Immunofluorescence and F-Actin localization

Cells were treated for 6 or 24 hours in μ*g* or kept at 1*g*. Samples were fixed with 4% paraformaldehyde for 10 min at 4 °C and then washed twice for 10 min with PBS. The cells were permeabilized for 30 min using PBS, 3% BSA, 0.1% Triton X-100, and then incubated at 4 °C for 12 hours with primary antibodies polyclonal anti-Fascin 1 (Santa Cruz Biotechnology, Santa Cruz, CA, USA, sc-28265) or monoclonal anti-α-Tubulin (Sigma-Aldrich, T5168), both diluted in PBS, 3% BSA. The cells were incubated for 1 hour at 25 °C with the appropriate FITC–conjugated secondary antibodies. For F-actin visualization, cells were fixed and permeabilized with cold Ethanol/Acetone 1:1 for 10 min at 4 °C. After rinsing in PBS, cells were incubated with TRITC-Phalloidin (Sigma-Aldrich, P1951) for 25 min in the dark and then washed in PBS. TOPRO-3 reagent was used for nuclei staining. Laser lines were 488, 543 and 633 nm for FITC, TRITC and TOPRO-3 excitation respectively. The images were scanned under a 20X objective or 40X oil immersion objective. Three independent experiments were performed.

### Confocal microscopy quantitative analysis

In order to perform quantitative analysis of fascin fluorescence, optical spatial series, each composed of 10/14 optical sections with a step size of 2 μm, were performed. 30 equivalent sized regions (Regions Of Interest; ROI), randomly drawn both in control and μg series, were analyzed. The ROI fluorescence intensity was determined by the Leica Confocal software using the maximum amplitude (MAF, Max Amplitude Fluorescence) of fluorescence parameter. To evaluate mitotic cells, both mitotic spindles and midbodies were counted along the entire z-axis of each optical spatial series performed both in control and in μg treated cells. We analyzed a total of 47 optical sections in the area where cells are confluent and 86 optical sections in the region of the scratch. Three independent experiments were performed.

### Western Blot

Cells were treated for 24 hours in μ*g* or kept at 1*g* and total proteins were collected, quantified, loaded and blotted as described^40^. Target proteins were marked with the following antibodies: polyclonal anti-E-cadherin (sc-7870), monoclonal anti-vimentin (sc-373717) and polyclonal anti-Fascin 1 (sc-28265) (all purchased from Santa Cruz Biotechnology), monoclonal anti- E-cadherin C-terminal end (E-cadherin FR) (BD Transduction Laboratories^TM^, San Diego, CA, USA, 610181), monoclonal anti-Snail1 (Cell Signaling Technology, Beverly, MA, USA, #3879), polyclonal anti-α-SMA (ab5694), polyclonal anti-MMP-1 N-terminal end active (ab38929) and polyclonal anti-MMP-3 N-terminal end (ab137741) (all purchased from Abcam, Cambridge, UK) and monoclonal anti-β-actin (Sigma-Aldrich, A2547). Antigens were detected with enhanced chemoluminescence kit (Amersham Biosciences, Little Chalfont Buckingamshire, UK), according to the manufacturer’s instructions. All Western blot images were acquired and analyzed through Imaging Fluor S densitometer (Bio-Rad, Hercules, CA, USA). Three independent experiments were performed.

### MMPs gelatin zymography

The enzymatic activities of *MMP2* and *MMP9* were determined by gelatin zymography. Briefly, conditioned media of μg and 1g cells were prepared with standard SDS–polyacrylamide gel loading buffer containing 0.01% SDS without β-mercaptoethanol and not boiled before loading. Then, prepared samples were subjected to electrophoresis with 12% SDS–PAGE containing 1% gelatin. After electrophoresis, gels were washed twice with distilled water containing 2.5% Triton X-100 for 30 min at 25 °C to remove SDS and then incubated in collagenase buffer (0.5 M Tris-HCl pH 7.5, 50 mM CaCl_2_ and 2 M NaCl) o.n. at 37 °C, stained with Coomassie brilliant blue R-250 and destained with destaining solution (30% methanol, 10% acetic acid, and 60% water). Three independent experiments were performed.

### Primers

The amplification of the cDNA fragments of Snail1, Snail2, *ZEB2*, N-cadherin, *MMP1*, *MMP2*, *MMP3*, *MMP9* expression and for the ribosomal 18S RNA housekeeping gene was obtained using oligonucleotide primers chosen through the online tool Primer-BLAST^42^ and purchased from Invitrogen (Invitrogen, Carlsbad, CA, USA). Primer list and characteristics are reported in Supplementary Table S1. For each primer pair, we performed no-template control which produced negligible signals.

### RNA extraction and cDNA synthesis

RNA was extracted using the Quick-RNA™ MiniPrep (Zymo Research, Irvine, CA, USA) according to manufacturer’s instructions. Each sample was treated, quantified and 1 μ*g* was used to reverse transcription using iScript cDNA synthesis kit (Bio-Rad) with thermal cycling programme as follows: 25 °C for 5 min, followed by 46 °C for 20 min and 95 °C for 1 min, as reported^36^.

### PCR amplification and real-time quantitation

RT-PCR was carried out in 96-well plate using iQ SYBR Green Supermix (Bio-Rad) and the iCycler Real-Time Detection System (iQ5; Bio-Rad). The thermal cycling programme was performed as follow: an initial denaturation step at 95 °C for 3 min, followed by 40 cycles at 95 °C for 10 s and 60 °C for 30 s. Melting curves were performed for each primer pair. The relative quantification of gene expression was assessed by comparative threshold cycle method (2^−DDCT^), including normalization of the gene expression to ribosomal 18S RNA, whose expression remains stable in all experimental conditions. All experiments have been done in triplicate and are reported as mean ± SD.

### Statistical analysis

Data were expressed as mean ± SD. Data were statistically analyzed with unpaired two-tailed Student’s *t*-test. Differences were considered significant at the level of p < 0.05. Statistical analysis was performed by using GraphPad Instat software (GraphPad Software, Inc.; San Diego, CA, USA).

## Electronic supplementary material

Supplementary Figure S1, S2, Supplementary Table S1
