# Supplementary material for: Simulated microgravity triggers epithelial mesenchymal transition in human keratinocytes
Source: Sci Rep. 2017 Apr 3;7:538. doi: 10.1038/s41598-017-00602-0 (PMC5428850; doi:10.1038/s41598-017-00602-0)
Supplement: Supplementary file 1 — Supplementary Figure S1, S2, Supplementary Table S1 [file 41598_2017_602_MOESM1_ESM.pdf]

Simulated microgravity triggers epithelial mesenchymal transition in human keratinocytes.

Danilo Ranieri*§1, Sara Proietti§1 , Simona Dinicola[1], Maria Grazia Masiello[1], Benedetta Rosato[1], Giulia Ricci[2], Alessandra Cucina[3], Angela Catizone[4], Mariano Bizzarri[5] and Maria Rosaria Torrisi[1]

[1]Dipartimento di Medicina Clinica e Molecolare, Sapienza Università di Roma, Italy

[2] Dipartimento di Medicina Sperimentale, Seconda Università di Napoli, Italy

[3]Dipartimento di Chirurgia "P. Valdoni", Sapienza Università di Roma, Italy

[4]Dipartimento di Scienze Anatomiche, Istologiche, Medico Legali e dell'Apparato Locomotore, Sapienza Università di Roma, Italy

[5]Dipartimento di Medicina Sperimentale, Sapienza Università di Roma, Italy

§ These authors contributed equally to this work.

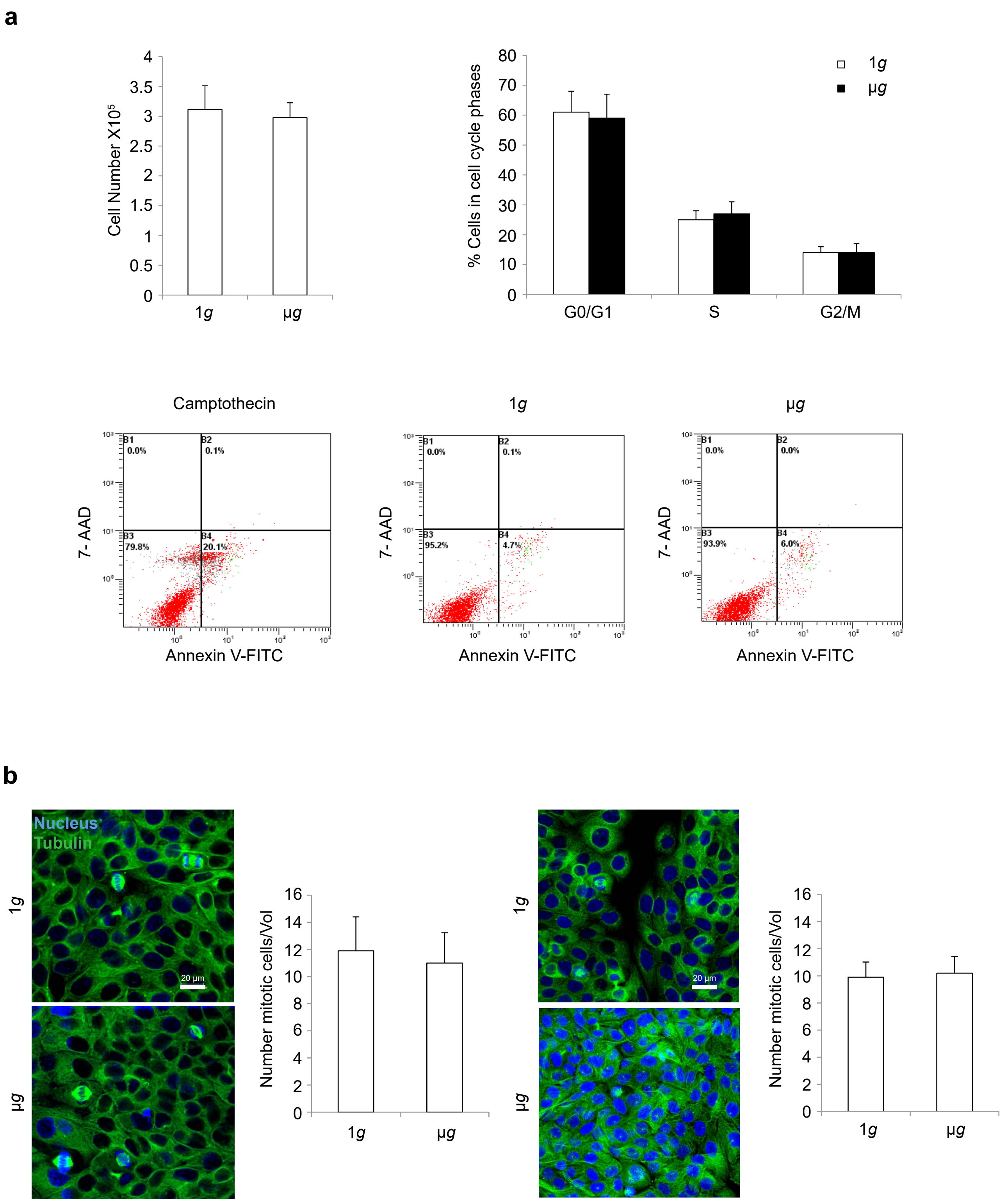

Supplementary Figure S1. Simuated microgravity does not affect cell proliferation or apoptosis. (**a**) Proliferation assay, cytofluorimetric analysis of the percentage of cell cycle phase distribution and Annexin V cytofluorimetric assay using camptothecin treatment as positive control for apoptosis were performed in HaCaT cells exposed to simulated microgravity for 24 hours (μ*g*) or kept at 1*g*. No significant differences in cell number increase (top left chart), in cell cycle phase distribution (top right chart) or in apoptotic rate are found in μ*g* cells respect to control cells (bottom panels). Results are expressed as mean ± SD from three different experiments in duplicate. (**b**) Immunofluorescence analysis using anti-β tubulin antibody in μ*g* or 1*g* HaCaT cells. Nuclei were stained with TOPRO-3. Mitotic spindles and midbodies count shows no significant differences in the number of mitotic cells/fields in μ*g* cells respect to control cells. Results are expressed as mean ± SD from three different experiments.

a

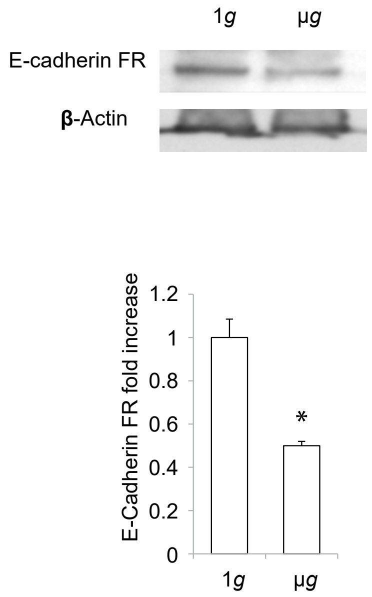

b

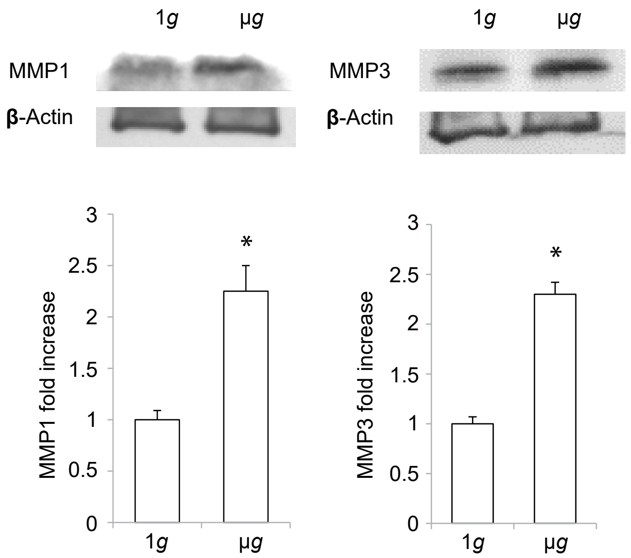

Supplementary Figure S2. Simulated microgravity treatment induces metalloproteases and E-cadherine fragment (FR) protein expression in HaCaT cells. Cells were exposed to μ*g* or kept at 1*g* for 24 hours. Western blot analysis shows a reduction of E-cadherin FR (**a**) and an increase of MMP1 and MMP3 protein levels (**b**) in simulated microgravity-treated cells compared to cells kept at 1*g*. The equal loading was assessed using anti-β actin antibody. For the densitometric analysis, the values from three independent experiments were normalized, expressed as fold increase and reported in graph as mean values ± SD. Student's *t* test was performed and significance levels have been defined as follows: * $p<0.05$ vs 1*g* cells.

Supplementary Table S1. Primers used for target and housekeeping genes.

Gene	Primer sequence (5'-3')<br>Forward-Reverse	Lenght	T_m
Snail1	5'-GCTGCAGGACTCTAATCCAGA-'3<br>5'-ATCTCCGGAGGTGGGATG-3'	21<br>18	58.24<br>57.70
Snail2	5'-TGGTTGCTTCAAGGACACAT-'3<br>5'-AGCAAATGCTCTGTTGCAGTG-3'	20<br>21	58.60<br>59.20
ZEB2	5'-AAGCCAGGGACAGATCAGC-'3<br>5'-CCACACTCTGTGCATTTGAACT-3'	19<br>22	59.39<br>59.38
N-cadherin	5'-CTCCATGTGCCGGATAGC-'3<br>5'-CGATTTCACCAGAAGCCTCTAC-3'	18<br>22	57.63<br>58.81
MMP1	5'-ACTGAGAAAGAAGAC AAAGGCAAG-3'<br>5'-TGGGCTGCTTCATCACCTTC-3'	24<br>20	59.42<br>60.32
MMP2	5'- CCTGATGTCCAGCGAGTGG-3'<br>5'- TCTTCTTCACCTCATTGTATCTCCAG-3'	19<br>26	60.15<br>60.13
MMP3	5'- TGGACAAAGGATACAACAGGGAC-3'<br>5'- TGTGAGTGAGTGATAGAGTGGGT-3'	23<br>23	60.24<br>60.50
MMP9	5'-CGCGCTGGGCTTAGATCATT-3'<br>5'- GGGCGAGGACCATAGAGGT-3'	20<br>19	60.88<br>60.46
18s	5'-AACCAACCCGGTCAGCCCCT-3'<br>5'-TTCGAATGGGTCGTCGCCGC-3'	20<br>20	66.14<br>65.80
